# Microsurgical Reconstruction of the Ear and Temporal Region: Structural and Functional Considerations Including Hearing Rehabilitation—A Narrative Review

**DOI:** 10.3390/audiolres16020047

**Published:** 2026-03-22

**Authors:** Florin-Vlad Hodea, Eliza-Maria Bordeanu-Diaconescu, Andrei Cretu, Vladut-Alin Ratoiu, Cristian-Sorin Hariga, Cristian-Radu Jecan, Ioan Lascar, Andreea Grosu-Bularda

**Affiliations:** 1 Department 11, Discipline Plastic and Reconstructive Surgery, University of Medicine and Pharmacy Carol Davila, 050474 Bucharest, Romania; florin-vlad.hodea@drd.umfcd.ro (F.-V.H.);; 2 Clinic of Plastic Surgery and Reconstructive Microsurgery, Clinical Emergency Hospital of Bucharest, 014461 Bucharest, Romania

**Keywords:** auricular reconstruction, microtia, temporal region, microsurgical techniques, hearing rehabilitation

## Abstract

Reconstruction of the ear and temporal region presents unique challenges due to the complex anatomy of the lateral skull base and the need to restore both structural integrity and auditory function. Historically managed as separate entities, auricular reconstruction and hearing rehabilitation are increasingly approached in an integrated manner, supported by advances in microsurgical techniques and implantable hearing technologies. This narrative review synthesizes contemporary evidence on microsurgical reconstruction of the ear and temporal region in conjunction with hearing rehabilitation, analyzing a wide range of existing surgical techniques in an integrative manner. Reconstructive techniques discussed include local and regional flaps, free tissue transfer, auricular framework reconstruction using autologous cartilage or alloplastic materials, external auditory canal reconstruction, and subtotal petrosectomy. Hearing rehabilitation options reviewed encompass bone-anchored hearing systems, active and passive transcutaneous devices, middle ear implants, and cochlear implantation. Simultaneous reconstruction and implantation may reduce surgical burden and enable earlier hearing restoration in carefully selected patients, while staged approaches remain advantageous in complex or high-risk scenarios, particularly in the presence of chronic infection or extensive temporal bone surgery. Multidisciplinary collaboration, meticulous preoperative planning, and long-term follow-up are essential to optimize outcomes.

## 1. Introduction

The reconstruction of the ear and temporal region following congenital malformations, traumatic injuries or oncological resections presents unique challenges that demand both aesthetic restoration and functional hearing rehabilitation. Congenital conditions such as microtia and aural atresia affect approximately 1 in 6000–12,000 live births, while acquired defects from temporal bone trauma, chronic otitis media, cholesteatoma and malignancies necessitate complex reconstructive interventions [1,2,3]. Modern management strategies are based on a coordinated multidisciplinary approach that combines microsurgical reconstruction with advanced hearing rehabilitation technologies to optimize both form and function.

Recent advances in implantable hearing devices, such as bone-anchored hearing aids (BAHA, Ponto, Bonebridge), middle ear implants (Vibrant Soundbridge) and cochlear implants, have improved auditory rehabilitation for patients with conductive, mixed, or sensorineural hearing loss [4,5,6]. Concurrently, refinements in microsurgical techniques, including free tissue transfer, local flap reconstruction and subtotal petrosectomy approaches, have expanded reconstructive options for complex temporal defects [7,8].

Historically, auricular reconstruction and hearing rehabilitation were performed as separate, sequential procedures. Contemporary practice increasingly favors integrated approaches, with simultaneous or coordinated staging of reconstruction and implantation [2,3,4,9]. This evolution reflects improved understanding of surgical anatomy, device technology advances and recognition of patient-centered benefits, including reduced surgical burden and earlier functional restoration [10,11].

This narrative review aims to provide an integrated overview of contemporary strategies for microsurgical reconstruction of the ear and temporal region in conjunction with hearing rehabilitation. By synthesizing recent evidence, the article examines reconstructive techniques, implantable hearing modalities, clinical outcomes, and current areas of consensus and controversy. Particular emphasis is placed on decision-making regarding the timing of reconstruction and implantation, device selection in complex anatomical scenarios, and the multidisciplinary principles required to optimize both aesthetic restoration and functional auditory outcomes.

## 2. Methodology

This study was conducted as a narrative review designed to synthesize and critically evaluate the current literature on microsurgical reconstruction of the ear and temporal region with integrated hearing rehabilitation. The review focused on reconstructive strategies for congenital and acquired auricular and temporal defects, implantable hearing technologies, surgical timing (simultaneous versus staged approaches), and clinical outcomes in complex reconstructive scenarios. A comprehensive literature search was performed using major academic databases, including PubMed, Web of Science, and Google Scholar. The search strategy incorporated combinations of keywords such as auricular reconstruction, temporal bone reconstruction, microtia, aural atresia, subtotal petrosectomy, bone-anchored hearing devices, middle ear implants, cochlear implantation and hearing rehabilitation. This review was designed as a narrative review aimed at providing a conceptual and clinically oriented synthesis of the literature. Therefore, the literature search was intended to identify relevant studies rather than perform an exhaustive systematic selection.

Articles were initially screened based on title and abstract, followed by full-text review of relevant studies. Priority was given to original clinical studies, systematic reviews and well-documented case series addressing reconstructive techniques, hearing rehabilitation outcomes, complication profiles and timing strategies. Particular attention was paid to studies reporting integrated surgical approaches and multidisciplinary management. The review included publications available up to 2026.

## 3. Characterization of Composite Lesions Affecting the Auricular and Temporal Regions

The temporal region comprises a complex three-dimensional anatomical unit that includes the external ear, the external auditory canal, the middle ear, the temporal bone and the surrounding soft tissues. Reconstruction in this area requires precise anatomical knowledge and careful spatial planning, particularly when addressing combined soft-tissue and osseous defects. Vascular anatomy is a key determinant of reconstructive feasibility, with the superficial temporal artery and its branches representing the principal recipient vessels for microvascular free tissue transfer in the temporal region [8]. The temporoparietal fascia provides a reliable, well-vascularized tissue layer that is frequently used for local flap reconstruction and coverage of reconstructive frameworks or implants. In addition, the mastoid cortex offers adequate bone quality and thickness for the fixation of osseointegrated hearing devices, provided that local anatomy and prior pathology permit safe implantation [4,12].

Defects of the ear and temporal bone are classified into congenital and acquired defects, each presenting distinct anatomical, functional and reconstructive challenges, as depicted in Table 1.

Congenital anomalies, most commonly microtia and aural atresia, arise from developmental malformations of the external and middle ear and vary in severity from minor structural abnormalities to complete absence of the auricle and ear canal. These conditions are frequently associated with conductive hearing loss and can significantly affect speech, language, and neurocognitive development, particularly in children, making early auditory rehabilitation essential alongside aesthetic reconstruction [13]. Microtia and aural are commonly stratified by severity using classification systems such as that proposed by Weerda. Mild forms, corresponding to Grade I, are characterized by a largely recognizable auricle with preservation of key anatomical landmarks and a usually present, though occasionally stenotic, external auditory canal and are typically associated with mild to moderate conductive hearing loss. Intermediate deformities, classified as Grade II, show partial auricular development with disorganized cartilage architecture and a rudimentary or absent external auditory canal, resulting in more pronounced conductive impairment and increased reconstructive complexity. The most severe presentations, grouped as Grade III, involve a severely hypoplastic “peanut-shaped” auricular remnant or complete anotia, complete aural atresia and significant middle ear malformations, conditions in which canaloplasty is rarely feasible and implantable hearing rehabilitation becomes the preferred functional strategy [14]. Across this spectrum, patients frequently present with conductive hearing loss averaging 50–60 dB, which necessitates not only aesthetic reconstruction but also effective auditory rehabilitation. In clinical practice, the functional consequences extend beyond audiometric thresholds, as impaired speech perception, reduced sound localization, and difficulties in everyday communication are commonly observed, particularly in pediatric patients during critical periods of neurocognitive and language development [2,3,15].

In contrast, acquired defects result from trauma, chronic infection, cholesteatoma, or malignancy and often involve complex composite tissue loss affecting skin, soft tissue, bone and auditory structures. These cases are further complicated by scarring, inflammation, prior surgical intervention, or the need for oncologic resection, all of which may limit reconstructive options and compromise local tissue quality [1,6,16].

Trauma to the temporal region can result from direct blunt or penetrating injury and may involve the auricle, middle ear, adjacent inner ear structures and temporal bone fractures. Auricular injuries range from simple lacerations to complete avulsion, with reconstruction guided by the same principles applied in microtia repair, often utilizing autologous rib cartilage grafts or prosthetic devices. When trauma produces composite defects extending beyond the auricle, management must be individualized, addressing not only anatomical reconstruction but also restoration of function, aesthetic appearance, and soft tissue coverage [17,18]. In addition, the external ear’s exposed cartilaginous structure renders it particularly susceptible to burns, which carry a high risk of chondritis and structural compromise. Management of burned ears aims to prevent infection, preserve anatomical form and function and salvage as much native auricular tissue as possible. In severe cases, complete auricular loss occurs, which can impair sound localization and compromise the psychological well-being of affected patients. Reconstruction in burn patients is challenging and typically delayed until the wound bed is stable and free of infection [19,20].

Tumors affecting the temporal region may arise from the skin, external auditory canal, middle ear, temporal bone, parotid gland, cranial nerves, or intracranial structures and may be benign or malignant, as well as primary or secondary (metastatic) [21]. Malignant tumors lead to large excisions and reconstructive needs, often requiring the assistance of plastic surgeons.

The most frequent malignancies of the temporal region arise from the temporal skin and periauricular area, with basal cell carcinoma being the most common, followed by squamous cell carcinoma, which has a higher risk of deep invasion. Other cutaneous malignancies in this region include melanoma and Merkel cell carcinoma [22].

Malignant tumors of the external auditory canal are represented by squamous cell carcinoma, as the most frequent primary malignancy in this location, followed by tumors such as basal cell carcinoma extending from the periauricular skin, ceruminous adenocarcinoma, and adenoid cystic carcinoma arising from glandular structures. Owing to the confined anatomy of the temporal bone and the proximity to critical neurovascular structures, these tumors have a strong tendency for early bone invasion and spread along soft tissue and neural pathways. As a result, advanced-stage disease often necessitates extensive oncologic resection, including lateral or subtotal temporal bone resection, to achieve adequate margins and local disease control [23].

Middle ear and temporal bone tumors are represented by squamous cell carcinoma extending from the external auditory canal or the middle ear, rhabdomyosarcoma (pediatric), chondrosarcoma, osteosarcoma, and temporal bone metastases (from breast, lung, kidney or prostate cancer) [24].

Because of anatomic proximity, parotid tumors such as adenoid cystic carcinoma, mucoepidermoid carcinoma, squamous cell carcinoma, adenocarcinoma, or acinic cell carcinoma may involve the ear canal, mastoid, or skull base and often require “en bloc temporal-parotid resection”, ranging from simple external auditory canal resection, to mastoidectomy, lateral temporal bone resection, subtotal temporal bone resection and even temporal bone resection [25].

Refractory chronic otitis media and advanced cholesteatoma represent a distinct subset of inflammatory conditions in which repeated infection, persistent otorrhea, and progressive bone destruction render conventional surgical strategies insufficient. In such cases, subtotal petrosectomy with closure of the external auditory canal, obliteration of the Eustachian tube, and cavity obliteration is often required to eradicate disease and create a stable, infection-free environment [1,26]. Although this approach sacrifices the native sound conduction pathway, it establishes a controlled anatomical setting that is favorable for implantable hearing rehabilitation. By eliminating the chronically infected middle ear space and isolating the surgical cavity from the external environment, subtotal petrosectomy reduces the risk of postoperative infection and provides improved exposure and safety for simultaneous or staged implantation of hearing devices [1,26].

A variety of traumatic, infectious, neoplastic and iatrogenic causes are associated with subsequent facial nerve palsy. Traumatic injury of the nerve is most frequently due to temporal bone fractures. Longitudinal fractures may occasionally spare the nerve, causing delayed onset palsy, whereas transverse fractures carry a higher risk of immediate and complete facial paralysis. Trauma can also occur iatrogenically during middle ear, mastoid, or cochlear implant surgery, as well as from external blunt or penetrating injuries to the temporal or periauricular region [27,28]. In addition, infectious and inflammatory conditions can compromise the facial nerve through direct invasion or secondary inflammation. Acute otitis media may rarely cause facial nerve inflammation, while chronic suppurative otitis media and cholesteatoma can erode the temporal bone and directly involve the nerve [29,30]. Tumors of the temporal bone, external auditory canal, and parotid region can all lead to facial nerve lesions. Primary malignancies such as squamous cell carcinoma, adenoid cystic carcinoma and ceruminous adenocarcinoma can invade the facial nerve, while benign or malignant parotid tumors may compress or infiltrate the mastoid segment of the nerve. Skull base tumors, including vestibular schwannomas, facial nerve schwannomas, and meningiomas, may similarly compromise facial nerve function depending on their location. Iatrogenic injury during otologic or skull base surgery remains a significant risk factor for facial nerve palsy [31,32]. Procedures such as mastoidectomy, canaloplasty, cochlear implantation, and lateral or subtotal temporal bone resection for tumor excision may inadvertently damage the nerve [28].

The presence of composite lesions affecting the auricular and temporal regions can lead to hearing impairment due to several causes: dysfunctions in the mechanical conduction of the sound to the inner ear, bad perception of sound by sensory hair cells in the cochlea, impaired transmission of auditory signals through the cochlear nerve to the cochlear nuclei in the brainstem and affected sensory procession in the auditory cortex. Hearing loss resulting from pathology of the sound conduction apparatus, including the auricle, external auditory canal, tympanic membrane and the ossicular chain of the middle ear responsible for transmitting acoustic energy to the cochlea, is termed conductive hearing loss. Sensory hearing loss is defined as hearing impairment resulting from dysfunction of the cochlea. Neural hearing loss refers to impairment caused by pathology of the cochlear nerve. Central hearing loss arises from dysfunction within the central auditory pathways or the auditory cortex [33]. Based on pure tone audiometric thresholds, hearing loss may be categorized as mild at 20 to 35 dB, moderate at 35 to 50 dB, moderately severe at 50 to 65 dB, severe at 65 to 80 dB, profound at 80 to 95 dB and total at 95 dB or greater [34].

## 4. Microsurgical Reconstruction of the Ear and Temporal Region: Indications and Techniques

As therapeutic principles, microsurgical reconstruction of the ear and temporal region must be guided by a defect-oriented and functional restoration approach, recognizing the composite nature of these defects and the close interdependence between structural reconstruction and auditory function. Accurate three-dimensional assessment of tissue loss is essential, with reconstruction planned to re-establish a stable auricular framework that provides durable projection, contour, and mechanical support for hearing rehabilitation devices [35,36,37].

Preservation or reconstruction of the external auditory canal, when involved, is critical to functional outcome and should aim to maintain long-term patency and a reliable epithelial lining. Given the frequent presence of scarred, irradiated, or infected recipient beds, the use of well-vascularized tissue, often through microsurgical free flaps, represents an essential objective of reconstruction, improving wound healing, resistance to infection, and tolerance of cartilage frameworks, alloplastic frameworks, or osseointegrated hearing systems [38,39,40,41,42,43,44].

Extensive oncologic resection or scar formation following ear and lateral skull base surgery can result in deficits of skin and supporting soft tissue, often making primary closure difficult. In such situations, regional flaps represent a reliable reconstructive solution, with the temporoparietal fascia (TPF) flap being one of the most versatile options. The TFP flap has reliable vascularization from the superficial temporal artery, providing thin axial-pattern fascial and fasciocutaneous flaps, and its long vascular pedicle provides a wide arc of rotation, allowing flap transposition over distances of up to approximately 12 cm [45,46,47].

In addition, its flexibility and generous tissue supply make it especially effective for covering exposed bone, promoting epithelialization, and decreasing cavity size. These properties initially led to its clinical use in the reconstruction of auricular deformities, such as microtia, resurfacing exposed cartilage and secondary reconstruction of the auricle when the skin presents extensive scars. It may also help shorten healing time after a modified radical mastoidectomy and can be used in covering defects after temporal bone resection, surgery for malignant otitis externa and revision mastoid surgery [46,48,49].

The TPF flap has advantages in alloplastic microtia reconstruction, since total coverage of the porous polyethylene implant with a temporoparietal fascia flap significantly reduces the risk of implant extrusion [50,51].

TPF flap is also useful in managing cochlear implant extrusion since its rich vascularity and pliability allow reliable coverage of the exposed implant, while promoting healing and the wide arc of rotation facilitates access to the implant site. Importantly, the TPF flap is thin, so its use does not significantly increase scalp thickness, taking into account that excessive soft tissue bulk (generally >6 mm) can interfere with magnetic coupling of the external processor [49].

Reconstructive planning must explicitly anticipate current or future hearing rehabilitation, ensuring adequate soft-tissue thickness, vascularity and spatial configuration for safe device implantation. Meticulous assessment of facial nerve integrity is mandatory, with early integration of nerve repair or reanimation strategies when indicated. In complex cases, staged reconstruction is often preferable, allowing sequential optimization of soft tissue coverage, structural support, and auditory rehabilitation while minimizing complications. Ultimately, successful microsurgical reconstruction in this region relies on multidisciplinary collaboration and should be evaluated not only by anatomical restoration but also by functional recovery, including hearing outcomes, long-term stability and patient quality of life. Recent research has been conducted aiming to introduce the latest technological techniques for optimizing auricular region reconstruction [52,53,54,55,56,57].

### 4.1. Auricular Reconstruction

Auricular reconstruction for microtia continues to rely predominantly on autologous rib cartilage framework fabrication, most commonly delivered through multi-stage strategies derived from the Brent, Nagata and Firmin concepts, with technical variation driven by surgeon preference, available soft tissue, and the anticipated need for concomitant otologic procedures [15,58].

Although these techniques share the common principle of constructing a three-dimensional auricular framework from harvested costal cartilage, they differ primarily in the number of operative stages, framework design and management of the soft-tissue envelope [59,60,61].

The Brent technique, introduced in 1980 and refined through extensive clinical experience, is traditionally performed in three or four stages. The first stage involves fabrication and insertion of a carved costal cartilage framework beneath the postauricular skin pocket. Subsequent stages address lobule transposition, elevation of the auricular framework to achieve projection, and refinement of contour and tragal reconstruction. The staged approach allows progressive adaptation of the soft tissues and reduces the risk of skin compromise; however, it requires multiple operations and a longer reconstructive timeline [53,59,61,62].

In contrast, the Nagata technique, introduced in 1993, was designed to reduce the number of operative stages while improving anatomical definition. This method typically involves a two-stage reconstruction. The first stage of the Nagata technique involves harvesting costal cartilage, typically taken from the sixth, seventh, and eighth ribs. These cartilages provide enough material to create the structural components of the ear. Important structures that must be recreated include the helix, antihelix, superior and inferior crura, scapha, triangular fossa, concha, tragus, and lobule. The carved pieces are assembled and sutured together to form a stable auricular framework that mimics the natural contours of the ear. Once the framework is completed, a subcutaneous pocket is created at the site of the microtic ear. Any residual abnormal cartilage remnants are removed while preserving as much of the available skin as possible. The newly constructed cartilage framework is then inserted into this pocket. The overlying skin is carefully draped over the framework so that the detailed contours of the cartilage become visible through the skin. The second stage of the Nagata technique is usually performed approximately six months after the first operation. During this procedure, the reconstructed ear is elevated from the side of the head through a posterior incision. A cartilage wedge graft, often taken from the remaining rib cartilage, is placed behind the ear to maintain its projection and create a proper auriculocephalic angle. To provide adequate soft tissue coverage, a fascial flap, commonly the temporoparietal fascia or local mastoid fascia, is used to cover the posterior cartilage surface. A split-thickness skin graft, usually harvested from the scalp or groin, is then applied to cover the posterior aspect of the elevated ear. This stage completes the reconstruction by creating a natural ear position and stable projection [63,64,65].

The Firmin technique represents a further refinement of Nagata’s principles, emphasizing individualized framework design and improved positioning of the reconstructed auricle relative to the contralateral ear. Firmin introduced modifications that allow greater mobilization of the cartilage framework and more accurate adjustment of the auricular axis and projection during the elevation stage. These refinements also aim to produce a thinner posterior auricular surface and improved symmetry with the normal ear, thereby enhancing aesthetic outcomes [66,67].

In contemporary series, the reconstructive sequence is largely dictated by envelope management. When local skin is limited or tightly adherent, staged expansion of the postauricular region is used to create a thin, well-vascularized pocket that can accept a high-definition framework with reduced tension, followed by subsequent refinements to projection, lobule position, and definition. Recent outcome syntheses emphasize that the dominant morbidity profile of autologous reconstruction is not a single complication but a pattern that includes hematoma, framework exposure, infection and scar-related problems, with event rates influenced by technique-specific soft tissue handling, framework complexity, and institutional experience [68,69,70]. Comparative analyses of classic autologous techniques suggest that while headline complication categories may be broadly similar across approaches, the balance between refinement potential, early envelope risk, and revision patterns varies, reinforcing the value of technique selection based on local tissue conditions rather than a one-size-fits-all algorithm [71].

Because microtia is inherently a combined aesthetic–functional disorder, modern planning increasingly treats the auricular envelope as a shared resource for both reconstruction and hearing rehabilitation. This has made “soft-tissue preservation for future implantation” more than a general principle; it has become a practical surgical constraint that affects incision design, expander placement, and framework positioning, particularly when bone-conduction systems or middle ear implants are expected [4,9,72]. In this context, reconstructive success is not only framework survival and contour but also maintaining an implant-friendly corridor that avoids scarred, thinned, or previously expanded skin at the eventual device site. The recent microtia literature on integrated care explains that conflicts between reconstructive scars, temporoparietal fascial integrity, and implant positioning are preventable when implant pathways are anticipated early, either through coordinated simultaneous procedures or deliberately separated staging with “reconstruction-aware” incision planning [4,9,72].

The principles of auricular reconstruction in acquired defects are consistent with those applied in microtia; however, these cases typically require a staged reconstructive approach and increased attention to achieving stable, long-term outcomes.

Alloplastic reconstruction using porous polyethylene implants covered by a vascularized temporoparietal fascia flap has evolved into an established alternative, mainly because it can reduce donor-site morbidity and, in many centers, compress the reconstructive timeline by decreasing the number of stages [7]. The trade-off remains the material-specific vulnerability to exposure and infection, which continues to be the most clinically relevant failure mode in modern reviews of porous polyethylene frameworks, even when meticulous fascial coverage is achieved [73,74]. These considerations become sharper when the reconstructive plan also includes hearing implantation, because exposure risk and the need to protect both the framework and the device can constrain surgical sequencing and soft-tissue choices. As a result, current evidence supports porous polyethylene reconstruction as a valid option in appropriately selected patients and experienced hands, but it also supports the persistent emphasis on robust vascularized coverage and cautious patient selection, particularly when additional implant hardware is anticipated in the same anatomical territory [7].

### 4.2. Temporal Bone and External Auditory Canal Reconstruction

Reconstruction of the external auditory canal following atresia repair or oncological resection remains a technically demanding endeavor due to the necessity of re-establishing a patent, functional canal while preserving or restoring middle ear mechanics. Contemporary strategies most often employ transmastoid approaches, which provide direct access to the middle ear space and ossicular chain without extensive disruption of pinna or external soft tissues and have been validated in multiple surgical series for their ability to minimize inadvertent injury to the facial nerve, a structure that is frequently anteriorly displaced or anatomically distorted in congenital atresia and post-inflammatory situations [7,75]. Within this framework, tympanoplasty using autologous fascia grafts remains a cornerstone for reconstructing the tympanic membrane, providing a biological interface that integrates with residual middle ear mucosa and resists retraction and reperforation over time, while ossiculoplasty with partial or total ossicular replacement prostheses crafted from titanium or hydroxyapatite has become standard for restoring the sound conduction mechanism when native ossicles are hypoplastic or absent [7,76]. Outcome studies in congenital atresia populations suggest that judicious selection of prosthesis type and meticulous attention to prosthesis-footplate approximation correlate with improved long-term functional gains, although overall hearing results remain constrained by the intrinsic variability of inner ear reserve and Eustachian tube function in these patients [77].

In the oncologic setting, the reconstructive paradigm shifts in accordance with the extent of temporal bone resection dictated by tumor margins and local invasion. Lateral temporal bone resections that spare the otic capsule may be amenable to defect management with local flap coverage or split-thickness skin grafting when tissue loss is superficial and vascular supply is robust, recognizing that these superficial repairs must still accommodate future surveillance for recurrence and allow for appropriate auditory rehabilitation planning [15,78]. When disease necessitates more extensive resection, such as subtotal petrosectomy, the reconstructive challenge increases commensurately; obliteration of the resultant cavity with autologous fat or microvascular free tissue transfer becomes essential to eliminate dead space, reduce the risk of postoperative infection and provide durable soft-tissue coverage that accommodates subsequent hearing device placement or adjuvant therapy [15,79,80]. Emerging literature underscores the importance of integrating reconstructive and rehabilitative goals in oncologic temporal bone surgery, highlighting that successful palliation is not solely measured by local disease control but also by restoration of quality of life through maintenance of an external auditory canal that is structurally and functionally competent or creating a stable bed for implantable hearing solutions when native canal restoration is not feasible [80].

### 4.3. Free Flap Reconstruction in Lateral Temporal Defects

Free tissue transfer is of utmost importance when dealing with large defects of the lateral temporal region or the lateral skull base, such as after oncological resections or major trauma, as it serves multiple purposes. For example, such reconstructions can provide durable soft-tissue coverage over exposed dura mater or skull, fill in the space created by drilling in the temporal bone or petrosectomy, serve as a barrier against contamination in already infected areas or facilitate further radiotherapy. Given all these different situations, the literature does not define a single all-purpose flap but rather a small set of “workhorse” flaps. In scalp and temporal reconstruction, the most frequently used flaps are the anterolateral thigh (ALT) flap and the latissimus dorsi (LD) flap, but sometimes the radial forearm free flap (RFFF) or the rectus abdominis flap can also be used, usually depending on whether the flap is intended to be thin and pliable for simple coverage or bulky to obliterate a cavity [81,82].

The ALT flap is reported as an effective and safe option for lateral skull base defects after temporal bone malignancies as it can provide wide coverage, a long pedicle and adaptable volume for the irregular cavities in the temporal region whilst allowing for further adjustments if the shape becomes an issue in time [83]. Originally described by Song et al. in 1984, the ALT flap gained popularity because of its reliability, versatility, and large skin paddle, which can be harvested either as a fasciocutaneous or musculocutaneous flap [84]. One of its major advantages is the ability to adjust thickness and volume, allowing the flap to be tailored to the requirements of different defects. In addition, the ALT flap can be harvested without changing the patient’s intraoperative position, enabling simultaneous work by the donor-site and recipient-site surgical teams and facilitating a single-stage reconstruction. Clinical experience has shown that the ALT flap is particularly useful in the reconstruction of extensive defects following subtotal petrosectomy or radical temporal bone surgery [83,84,85]. The ALT flap is based on perforators from the descending branch of the lateral circumflex femoral artery, which travel through the vastus lateralis muscle or between muscular septa before reaching the skin. During flap harvest, the surgeon identifies these perforators using Doppler or visual exploration and carefully dissects them to preserve their connection with the main vascular pedicle. Once the flap is elevated, it is transferred to the recipient site and connected to recipient vessels through microsurgical anastomosis [86,87]. The flap consistently provides a long vascular pedicle with vessels of adequate caliber, which allows flexibility in choosing recipient vessels and often eliminates the need for venous grafting. The flap can be harvested as a fasciocutaneous flap for thin coverage when contour preservation is important, or it can include a portion of the vastus lateralis muscle when bulk is needed to fill dead space, such as after temporal bone drilling or petrosectomy. In addition, the flap can be thinned during harvest to better match the thin skin of the temporal region [83,84,85].

The radial forearm free flap (RFFF) represents an important microsurgical option in the reconstruction of complex auricular defects, particularly in situations where traditional techniques such as autologous rib cartilage reconstruction or local fascial flaps cannot be used. In auricular reconstruction, the radial forearm flap is most commonly used in cases of severe trauma, near-total or total ear amputation, extensive soft tissue loss, or failed previous reconstructions, when the local tissues of the temporal region are scarred, irradiated, or insufficient for reconstruction [88,89]. The flap is valued for its thin, pliable skin, reliable vascular anatomy, and long vascular pedicle, which make it suitable for reconstructing delicate three-dimensional structures such as the external ear. Because the forearm skin closely mimics the thin soft tissues of the auricular region, the radial forearm flap allows precise contouring and adaptation to the anatomical requirements of the ear. A key concept in radial forearm flap-based auricular reconstruction is the technique of prelamination or prefabrication. During the first stage of the procedure, a structural framework, often made of porous polyethylene or occasionally cartilage, is inserted beneath the skin of the forearm. Over time, the surrounding tissues integrate with this framework, forming a composite flap with both soft tissue coverage and structural support. After this integration period, the flap containing the framework is harvested together with its vascular pedicle and transferred to the auricular region. Microsurgical anastomosis is then performed to connect the radial artery and accompanying veins with suitable recipient vessels in the head and neck. This staged approach allows the surgeon to construct the three-dimensional auricular architecture before transplantation, improving the final contour and structural stability of the reconstructed ear [89,90,91]. Despite these benefits, the radial forearm flap also presents limitations, particularly related to donor-site morbidity, since harvesting the flap usually leaves a forearm defect that requires coverage with a split-thickness skin graft and may result in visible scarring. Moreover, the procedure is technically demanding and requires advanced microsurgical expertise because harvesting the flap involves sacrifice of the radial artery [92].

In contrast, when the priority is the management of massive dead spaces or the salvaging of improper recipient beds, muscular flaps, such as the LD flap, should be taken into consideration, and the indication should be carefully weighed rather than remaining in an “LD by default” mindset [81,82].

The latissimus dorsi flap is supplied by the thoracodorsal artery and vein, branches of the subscapular vascular system, which provide a long and consistent pedicle suitable for microvascular anastomosis in free tissue transfer while also allowing the flap to be used as a pedicled flap in selected cases where its arc of rotation permits coverage of nearby defects, particularly in patients who are not good candidates for free flap reconstruction. The large surface area and well-vascularized muscle allow effective coverage of exposed bone or implants after procedures such as temporal bone resection or petrosectomy. In the auricular region, the flap can provide a stable vascularized bed for cartilage frameworks, alloplastic implants, or prosthetic rehabilitation. Its relatively thin and pliable muscle can adapt to the contours of the temporal and periauricular area while also providing sufficient volume to fill surgical cavities [93,94].

The real problem with the temporal region is the choice of recipient vessels, as it often influences the feasibility of the flap, the pedicle length and the risk of thrombosis. The superficial temporal pedicle is mostly used as a recipient for defects centered in the temporal fossa or the auriculotemporal scalp since they allow for a short pedicle and avoid opening the neck, which is especially useful in patients who have previously undergone treatments for head and neck malignancies [81,82]. Recent evidence comparing the superficial temporal vessels and other cervical recipients shows similar outcomes, keeping in mind that flap success is dependent on vessel caliber assessment, quality of blood flow and venous outflow options, rather than assuming that the superficial temporal vessels are always appropriate and sufficient [95]. This observation is valuable, especially in “frozen neck” patients, where vessel depletion and fibrosis due to radiotherapy and prior surgery make cervical vessels unreliable or even unavailable. In these cases, the superficial temporal vessels are constantly mentioned as a reliable alternative, including the use of anterograde and retrograde limbs to expand the options for arterial inflow or venous outflow [96]. This conclusion is equally reached in the more recent literature focusing only on “frozen neck” patients, assuming that pedicle dissection is thorough and the surgeon anticipates venous strategy from the beginning rather than adapting after flap harvesting [97]. This is the exact scenario where decision-making becomes most “useful”: not which flap is fashionable but whether the reconstructive plan survives the reality of the recipient-vessel.

The complications in free flap reconstruction of the temporal region are predictable, which makes it easier to counsel the patient and decide between different reconstructive options. Reintervention is mostly due to vascular issues, especially venous compromise, wound dehiscence at sites of previously irradiated or scarred skin, pre-existing infection in recipient beds and delayed contour problems leading to further revisions. As expected, there are risk factors that predispose patients to such complications, especially smoking, which has been associated with a higher risk for major complications in complex scalp reconstructions [98]. Other factors that must be taken into consideration, particularly in oncologic lateral skull base surgery, are the risk of cerebrospinal fluid leakage, the need for dura mater repair, prior radiotherapy and the need for postoperative surveillance. The success of lateral skull base reconstructions should be measured not only by the flap survival but also by the achievement of compartment separation in a stable manner and long-term wound healing in a hostile field [99]. Practically, these complications plead for a reconstructive algorithm that involves selecting well-vascularized tissue in high-risk recipient beds, conservative venous planning with alternate venous pathways as a readily available back-up plan and aiming for minimal tension and ischemia in tissue margins in patients with compromised skin.

### 4.4. Subtotal Petrosectomy Techniques

Subtotal petrosectomy (STP) is usually indicated in cases of chronic suppurative otitis media, temporal bone fractures with persistent CSF leak, or cholesteatoma or before cochlear implantation in complex cases [1,100]. The procedure refers to the removal of air cells from the temporal bone, followed by the closure of the external auditory canal and the tube of Eustachio and the obliteration of the resulting cavity with abdominal fat [100,101].

STP reduces the risk of infection for subsequent implantable devices by creating a “safe, dry ear” through the elimination of the communication between the middle ear and the external environment [1,102]. It improves surgical access and visibility for cochlear implantation, especially in patients with cochlear ossification, distorted anatomy from previous interventions or inner ear malformations [23,102]. The downside of the procedure is the permanent loss of conductive hearing if any residual hearing exists, which should be carefully taken into account when deciding which patients might benefit from electroacoustic stimulation [102,103].

## 5. Hearing Rehabilitation Modalities

Hearing rehabilitation represents a fundamental component of the management of patients with auricular and temporal bone defects. In many cases, reconstruction of the external ear and temporal structures alone cannot fully restore auditory function, particularly when the sound conduction pathway is absent or severely damaged. Therefore, modern treatment strategies integrate reconstructive surgery with implantable hearing technologies in order to achieve both structural restoration and functional auditory recovery.

The choice of hearing rehabilitation modality is closely related to the underlying pathology and the anatomical conditions created during reconstruction. Factors such as the presence or absence of the external auditory canal, the integrity of the middle ear structures, the thickness and vascularity of soft tissues and the availability of suitable bone for fixation all influence the selection and timing of implantable devices. Consequently, successful outcomes rely on coordinated planning between reconstructive surgeons and otologic specialists.

Contemporary literature consistently demonstrates meaningful audiological benefit across available hearing rehabilitation modalities, although reported outcomes vary depending on patient populations, underlying pathology, and the specific devices employed. Despite heterogeneity in study design and outcome measures, functional hearing improvement remains a reproducible finding across bone conduction devices, cochlear implants and middle ear implants.

### 5.1. Bone-Anchored Hearing Systems

In patients with conductive or mixed hearing loss, bone-anchored hearing systems represent a foundation of auditory rehabilitation as they bypass the external and middle ear and facilitate sound transmission through direct bone conduction. Current practice involves two major categories: transcutaneous systems (such as Bonebridge, Sophono and BAHA Attract) and percutaneous systems (such as BAHA and Ponto) [3,4,104,105].

Percutaneous systems are based on an abutment through the skin that provides direct mechanical coupling between the osseointegrated implant and the external sound processor. The Ponto system, for example, was developed after the introduction of Ponto surgery, a minimally invasive procedure that involves the use of a drill guide to perform a punch incision, minimizing soft tissue damage and improving aesthetic results [106]. A ten-year systematic review of clinical use of Ponto devices provides long-term data attesting to consistent audiological benefit and high satisfaction among patients, despite the recognized limitation of soft tissue complications around the percutaneous abutment [105].

Transcutaneous systems were conceived of to reduce infection risk and improve cosmetic outcomes by eliminating the skin-penetrating component. The Bonebridge system, an active transcutaneous device, uses electromagnetic coupling to an implanted floating mass transducer in the temporal bone in order to transmit sound [3,4,5,107]. BAHA Attract and Sophono, which are passive transcutaneous devices, transmit sound via magnetic coupling across intact skin, with similar advantages in terms of cosmetic outcomes and skin integrity [3,108].

Clinical data comparing various transcutaneous systems shows comparable functional outcomes. A recent study on simultaneous implantation and auricular reconstruction in a pediatric population compared Bonebridge, Sophono and BAHA Attract and found no significant difference in hearing gain between these devices. Significant improvements in both hearing thresholds and speech recognition were observed when using the devices compared with unaided conditions. In terms of complications, all groups showed minor complications that could be managed conservatively, emphasizing the safety and effectiveness of these systems in patients who are appropriately selected [3].

### 5.2. Middle Ear Implants

The Vibrant Soundbridge (VSB) is an active middle ear implant that directly stimulates the structures of the middle ear through a floating mass transducer (FMT) attached to the ossicular chain or round window. VSB is indicated for conductive or mixed hearing loss when conventional hearing aids are contraindicated or poorly tolerated [9,10,109].

Recently, VSB implantation timing was adjusted, with multiple studies reporting successful implantation before auricular reconstruction in microtia patients [9,10]. Ueda et al. described a new incision design, approximately 5 cm posterior to the temporomandibular joint, at least 2 cm from the anticipated auricular reconstruction site, allowing the implantation of the VSB, avoiding two-stage auricular reconstruction. This technique facilitated early hearing rehabilitation while maintaining stable auricular contours postoperatively [9].

In select cases, the VSB is superior to bone conduction devices, providing potentially better sound quality for mixed hearing losses and avoidance of transcutaneous attenuation. However, VSB can only work if the anatomy of the middle ear is intact or there is a suitable access to the round window, which is not feasible in patients with severe atresia or who have undergone petrosectomy [110].

### 5.3. Cochlear Implantation

In patients with severe to profound sensorineural hearing loss or, more recently, even single-sided deafness and other complex temporal bone pathologies, cochlear implantation is the definitive solution for auditory rehabilitation [1,8,16,26,111]. Even in more complicated reconstructive scenarios, such as chronically infected ears or anatomical distortions of the temporal bones, cochlear implantation can be an option due to advances in surgical technique, implant design and preoperative planning. Cochlear implantation in combination with subtotal petrosectomy is an effective way to address both disease eradication and auditory rehabilitation in patients with chronic ear disease, either as a single-stage or a staged procedure [1,101,102]. Zhang et al. reported on a cohort of 37 patients undergoing subtotal petrosectomy with either osseointegrated hearing rehabilitation or cochlear implantation, with significant improvements among those with cochlear implants, whose speech recognition thresholds improved from 80 ± 21 dB to 31 ± 9 dB; AzBio scores increased from 11% to 43% and consonant–nucleus–consonant scores rose from 6% to 47% [1]. Despite these remarkable outcomes, 16% of cases scheduled for a single-stage approach were complicated by infection or wound dehiscence, requiring revision surgery, highlighting why careful patient selection and personalized decision-making are important when deciding between simultaneous versus staged procedures [1]. Additional technical challenges have been cited in cases of complex temporal bone injury, especially when temporal bone fractures are complicated by cochlear ossification [26,111]. Thus, Frisina et al. described a comprehensive strategy combining subtotal petrosectomy with advanced preoperative planning using OTOPLAN software for electrode selection and intraoperative use of an auditory nerve test system to confirm neural responsiveness prior to definitive implant placement. This approach reduced the risk of implantation failure in highly modified cochlear anatomy and illustrates the growing impact of digital planning and intraoperative functional assessment in complex cochlear implantation [26]. An emerging and particularly challenging indication is cochlear implantation after resection of intralabyrinthine schwannoma, where the balance between oncologic control, auditory rehabilitation, and long-term surveillance remains delicate [8,16]. Häussler et al. evaluated outcomes in ten patients undergoing combined schwannoma resection and cochlear implantation, reporting meaningful speech perception improvement in half of the cohort [16]. However, outcomes were not homogeneous, with limited benefit to some patients, and postoperative surveillance through MRI imagery was complicated by implant-related artifacts [8,16].

In patients who refuse surgery or have contraindications to implantation, conventional bone conduction hearing aids and air conduction devices are still important options. BiCROS (bilateral contralateral routing of signal) systems provide an alternative for single-sided deafness, despite the fact that they do not restore true binaural hearing [8].

## 6. Integrated Surgical Approaches

### 6.1. Simultaneous Reconstruction and Implantation

Simultaneous reconstruction and hearing device implantation has gained increasing acceptance in recent years as it offers several potential advantages, including a reduced overall surgical burden, earlier functional hearing restoration and improved cost-effectiveness when compared with staged treatment strategies [3,4,9,10,112]. Advances in surgical planning, implant design, and multidisciplinary coordination have contributed to the growing feasibility of combined procedures in appropriately selected patients.

Multiple studies have reported favorable outcomes following simultaneous auricular reconstruction and bone-anchored hearing device implantation [3,4,112,113]. Wang et al. described concomitant total auricular reconstruction with Bonebridge implantation using a retrosigmoid sinus approach in a cohort of 15 patients involving 28 reconstructed ears. This surgical strategy was specifically designed to avoid interference with the reconstructed auricular flap while achieving significant audiological improvement and significant enhancement of speech recognition in both quiet and noisy environments. Hearing outcomes remained stable over a follow-up period of 21 to 35 months and aesthetic results were reported as satisfactory in all cases [4].

Comparable findings have been reported in pediatric populations. Chen et al. evaluated simultaneous bone conduction device implantation and auricular reconstruction in children with microtia, directly comparing Bonebridge, Sophono, and BAHA Attract systems. The combined procedures resulted in significant improvements in hearing performance across all device groups, with only minor complications such as skin reactions, hematoma, and pressure erythema, all of which were managed conservatively. Importantly, no major adverse events were observed, supporting the overall safety and feasibility of simultaneous approaches in carefully selected pediatric patients [3].

In cases of severe auricular malformation, corresponding to Weerda type III deformities where autologous auricular reconstruction is not feasible, simultaneous implantation of epithesis anchors and Bonebridge has been proposed as an alternative strategy for combined aesthetic and functional rehabilitation. Patient-reported outcomes indicated high levels of satisfaction with both audiological performance and cosmetic appearance, further supporting the role of this combined approach in selected severe deformities [112,113].

### 6.2. Staged Procedures

Staged approaches separate reconstruction and hearing device implantation into distinct procedures and continue to represent an important strategy in the management of auricular malformations and hearing rehabilitation, particularly in complex or higher-risk cases. By decoupling the reconstructive and audiological components, staged protocols may reduce the complexity of individual operations and allow each step to be optimized independently, while preserving flexibility in overall treatment planning [4,9,10,72].

One increasingly adopted staged strategy involves hearing device implantation prior to auricular reconstruction, with the aim of achieving early functional rehabilitation while maintaining the integrity of subsequent aesthetic procedures [9,10,72]. Yoshimura et al. reported the first successful Vibrant Soundbridge implantation performed before auriculoplasty in a child with unilateral microtia and aural atresia, demonstrating meaningful hearing improvement under safe operative conditions [10]. Building on this concept, Ueda et al. described a novel incision design that deliberately avoided the future auricular reconstruction field, thereby preventing interference with subsequent two-stage auriculoplasty and preserving reconstructive options [9]. This sequencing enables earlier auditory input during critical developmental periods, reduces the risk of device damage during later reconstructive stages, and maintains surgical flexibility for optimal auricular framework positioning, provided that careful preoperative planning is undertaken to avoid compromising either procedure [9,10].

Traditional staged pathways involve implantation of hearing devices after completion of auricular reconstruction and remain widely practiced. In this context, Wang et al. reported outcomes following Bonebridge implantation using a retrosigmoid approach performed either during the third stage of auricular reconstruction or after completion of all reconstructive stages. This strategy resulted in significant audiological improvement, as well as excellent speech recognition performance in both quiet and noisy environments. Complication rates were low, with only a single delayed hematoma reported among 43 cases, which was successfully managed by aspiration, and no additional adverse events were observed [4].

## 7. Complications and Management

### 7.1. Surgical Complications

Depending on the type of procedure performed and the timing strategy that is chosen, surgical complications can vary, but the most significant complication from a clinical standpoint remains infection, particularly in combined approaches.

Zhang et al. reported that 6 out of 37 patients (16%) who had single-stage subtotal petrosectomy with implantation of hearing devices underwent revision surgery as a result of either infection or wound dehiscence, which is notably more frequent than in staged procedures. This observation supports the concept of a higher risk when cavity obliteration is combined with implantation in a single-stage setting [1]. In this study, revision was not necessary for any patients receiving osseointegrated hearing implants, as opposed to 16% of cochlear implant cases, reflecting disparities in device size, extent of surgical dissection, or patient selection [1].

In contrast, substantially lower complication rates have been reported in studies evaluating simultaneous auricular reconstruction combined with a bone-anchored hearing device [3,4]. Wang et al. observed only one delayed hematoma among 43 cases of Bonebridge implantation performed after auricular reconstruction, which could be resolved successfully with aspiration and without further sequelae [4]. Similarly, only minor complications were reported by Chen et al., including skin reactions, hematoma and pressure erythema, all of which could be managed conservatively [3].

Hematoma is a relatively common complication in these settings. However, it is a minor complication that does not require anything more than conservative treatment or simple aspiration [3,4]. In the pediatric microtia cohort reported by Chen et al., hematoma was among the minor postoperative events occurring after simultaneous bone conduction device implantation and auricular reconstruction, with no cases requiring any surgical reintervention [3]. Regardless of the type of reconstruction, implant extrusion remains a theoretical concern, particularly for porous polyethylene implants and hearing devices [114].

Complications related to the recipient site include cerebrospinal fluid leak, dural exposure, meningitis, as well as injury to the sigmoid sinus or jugular bulb, particularly in cases involving subtotal petrosectomy or extensive drilling of the temporal bone [115]. CSF leaks usually result from inadvertent dural violation or insufficient sealing of the otic capsule and are most often managed by multilayer closure techniques, the use of fibrin sealants, lumbar drainage, or, in persistent cases, revision surgery [116]. Although vascular injury is rare, it may lead to significant intraoperative bleeding and requires immediate recognition and adequate surgical control.

Facial nerve dysfunction represents one of the most critical complications in this anatomical region. Injury to the facial nerve may occur as a result of direct trauma, thermal damage during drilling, traction, or ischemia, especially during tumor resection or cochlear implantation in malformed or previously operated temporal bones [117]. Facial palsy may be evident immediately after surgery, suggesting intraoperative nerve injury, or may develop in a delayed manner, most commonly due to inflammatory or viral mechanisms [118]. When a facial nerve lesion is identified intraoperatively, immediate repair is recommended. Primary end-to-end anastomosis should be performed whenever feasible; otherwise, interposition nerve grafting using the greater auricular or sural nerve is indicated in order to optimize functional recovery [117]. In cases of delayed or incomplete recovery, secondary facial reanimation procedures may be required.

In patients undergoing free flap reconstruction of the ear or temporal region, flap-related complications may include venous congestion, arterial thrombosis, partial or total flap loss, fat necrosis and contour irregularities [119,120]. Although total flap failure is uncommon in experienced centers, close postoperative monitoring remains essential, as early surgical re-exploration may allow salvage of compromised flaps in selected cases. Minor complications, such as marginal flap necrosis or wound dehiscence, are encountered more frequently and are generally managed with local wound care or limited secondary revision procedures [119].

Donor-site complications should also be considered, particularly when radial forearm, anterolateral thigh, or scapular flaps are harvested. These may include hematoma, seroma, wound infection, delayed healing, sensory disturbances and aesthetic concerns at the donor site. Although donor-site morbidity is usually limited, it may have a negative impact on patient-reported outcomes and overall quality of life, highlighting the importance of careful flap selection and meticulous closure techniques [121].

### 7.2. Device-Related Complications

Device-related complications are generally influenced by the design of the chosen hearing system. Percutaneous bone-anchored hearing systems have the implicit risk of skin reactions at the site of abutment placement [105,122,123]. Leone et al. reported adverse skin reactions in one of nine pediatric patients (11.11%) undergoing minimally invasive Ponto surgery, with a mean follow-up of 11.4 months [123].

By eliminating the percutaneous component, transcutaneous systems manage to avoid soft-tissue complications [3,4]. While these systems reduce the risk of skin infection, pressure-related erythema secondary to magnetic coupling may occur and is generally managed with temporary device discontinuation or adjustment of magnet strength [3].

For cochlear implantation, another complication can be the incomplete insertion of the electrode, especially in cases with cochlear ossification, anatomical distortion or intralabyrinthine pathology [8,16]. Franchella et al. reported incomplete electrode insertion in patients undergoing cochlear implantation following intralabyrinthine schwannoma resection, which may decrease final audiological outcomes [8].

Moreover, cochlear implants can interfere with MRI readings, complicating postoperative imaging follow-up for pathologies such as intralabyrinthine schwannoma, a disadvantage that has to be carefully taken into account when opting for implantation rather than observation strategies in tumor-related disease [8,16].

### 7.3. Revision Surgery

Revision rates differ substantially according to both the surgical approach and the type of hearing device implanted. Zhang et al. reported a revision rate of 16% in patients undergoing single-stage subtotal petrosectomy with cochlear implantation, compared with a 0% revision rate in those receiving osseointegrated hearing implants, suggesting that device characteristics, procedural complexity, and underlying patient factors significantly influence revision risk [1]. Bone-anchored hearing systems may require revision for reasons such as fixture failure, inadequate osseointegration, or device upgrade; however, contemporary series employing modern implant designs and refined surgical techniques have demonstrated low revision rates overall [3,105,123].

Table 2 depicts the complications encountered in relation to microsurgical ear and temporal region reconstruction with hearing rehabilitation.

## 8. Discussions

The central concept emerging from this review is that modern management of auricular and temporal defects should not treat reconstruction and hearing rehabilitation as separate therapeutic pathways. Instead, both components must be integrated within a single reconstructive strategy aimed at restoring anatomical structure while simultaneously enabling effective auditory rehabilitation.

Head and neck reconstruction has evolved significantly over recent decades. While diverse flap techniques have been used for centuries, their early application was limited by an incomplete understanding of vascular anatomy. Advances in anatomical knowledge have substantially expanded reconstructive possibilities. Modern head and neck reconstruction is grounded in two core objectives: restoration of function and restoration of aesthetic form. Achieving optimal results in primary reconstruction is essential, alongside the strategic use of secondary procedures to further enhance functional and aesthetic outcomes, which should be tailored to the specific anatomical structures involved [124,125].

A particular anatomical region is represented by the ear and temporal area, which are characterized by exceptional anatomical and functional complexity. Beyond the restoration of auditory function, successful reconstruction must address multiple structural, protective, neural, sensory and psychosocial factors that directly influence long-term outcomes and patient adherence. Current evidence regarding reconstructive strategies of the ear and temporal region is largely derived from single-center case series, highlighting the need for high-quality comparative studies to clarify optimal timing strategies, device selection criteria and surgical approaches for specific patient populations [1,3,126,127,128].

Longer-term follow-up data are also required to evaluate device survival, revision rates, audiological stability and quality-of-life outcomes, as most published studies report follow-up periods limited to one to three years [3,8,16].

Dedicated pediatric research is particularly important given developmental considerations, device longevity requirements and the likelihood of multiple revisions over a patient’s lifetime. Furthermore, well-designed cost-effectiveness studies comparing simultaneous and staged surgical strategies, as well as different implant technologies, would provide valuable guidance for clinical decision-making and healthcare resource allocation [3,129,130,131].

Current evidence supports a multidisciplinary approach involving plastic surgery, otolaryngology and audiology to optimize both aesthetic and functional outcomes. Timing of reconstruction and implantation should be individualized based on patient age, anatomical considerations, disease complexity, surgeon experience and patient or family preferences rather than rigid protocols [11,69].

Simultaneous reconstruction and implantation can be safely performed in carefully selected patients by experienced teams, whereas staged approaches should be considered in cases involving chronic infection, complex pathology or elevated surgical risk [1,3,132].

Comprehensive preoperative planning remains fundamental to optimizing device selection and placement, reducing surgical risk and minimizing postoperative complications. When available, advanced three-dimensional imaging and virtual planning tools provide valuable anatomical detail, facilitating accurate assessment of spatial relationships and surgical feasibility. Equally critical is thorough preoperative patient counseling, including discussion of realistic functional and aesthetic expectations, potential intraoperative and long-term complications and the likelihood of revision procedures over time. Furthermore, systematic long-term follow-up is essential to monitor device performance, assess durability, detect delayed complications and ensure sustained functional benefit and patient adherence [8,26,105,133].

Aesthetic outcomes following integrated reconstruction and implantation approaches are generally favorable, with studies reporting satisfying cosmetic results [134,135,136]. Wang et al. noted satisfying aesthetic outcomes in all patients undergoing simultaneous auricular reconstruction and Bonebridge implantation [4]. Ueda et al. demonstrated that VSB implantation prior to auricular reconstruction maintained stable auricular contours postoperatively, with no compromise to reconstructed ear appearance [9]. For patients with severe malformations requiring epithesis rather than autologous reconstruction, simultaneous implantation of epithesis anchors and Bonebridge achieved satisfactory cosmetic outcomes alongside functional hearing improvement [112].

Beyond audiometric measures, hearing rehabilitation significantly improves quality of life for patients with auricular and temporal defects [69,137]. Patients with aural atresia experience significant improvements in quality of life following combined plastic reconstructive and audiological rehabilitation. By simultaneously addressing anatomical restoration and auditory function, this integrated approach enhances communication ability, social interaction, and psychosocial well-being, leading to more durable functional outcomes and improved long-term patient satisfaction [69,138]. Patients with minimally invasive Ponto surgery reported high satisfaction, with improved hearing performance enabling better communication and social interaction [122].

The psychological impact of combined aesthetic and functional rehabilitation extends beyond hearing improvement alone, addressing body image concerns and social stigma associated with visible ear malformations. Qualitative research increasingly emphasizes patient and family experience, using patient-centered methods to improve communication and optimize reconstructive outcomes [11,69,139].

### 8.1. Overview of Current Indications

Table 3 summarizes the main areas of consensus and unresolved controversies reported in the current literature regarding the reconstructive strategies of the auricular region and auditory rehabilitation.

The optimal timing of auricular reconstruction and hearing device implantation remains an area of ongoing debate, with both simultaneous and staged strategies supported by distinct advantages and limitations. Proponents of simultaneous approaches emphasize the potential to reduce the cumulative surgical burden and overall exposure to anesthesia while also enabling earlier functional hearing rehabilitation and consolidating costs through a single operative episode. In experienced centers, several series have reported that combined procedures do not significantly increase operative time or perioperative risk and demonstrated stable audiological and aesthetic outcomes across both adult and pediatric populations [2,4,112]. In contrast, staged approaches continue to be advocated in scenarios when surgical complexity, infection risk, or anatomical uncertainty may outweigh the benefits of a single-stage strategy. By separating reconstruction and implantation into distinct procedures, staged protocols may reduce the technical demands of individual operations and allow each component to be optimized independently. This flexibility can be particularly relevant in cases requiring cochlear implantation, where concerns regarding postoperative infection, wound healing and cavity management are more pronounced or when the initial surgical outcome necessitates modification of the subsequent treatment plan [1,9,10]. Evidence supporting a cautious approach to simultaneity in selected high-risk settings is illustrated by the findings of Zhang et al., who reported that 16% of patients undergoing planned single-stage subtotal petrosectomy combined with cochlear implantation required revision surgery for infection or wound breakdown [1]. This revision rate was notably higher than that observed in staged procedures and highlights the vulnerability of combined approaches when extensive cavity obliteration is performed in conjunction with the implantation of large electronic devices. These data underscore the importance of patient-specific risk assessment rather than routine adoption of a single-stage protocol in all cases [1].

Despite these differing perspectives, there is broad agreement within the contemporary literature that both simultaneous and staged strategies can achieve excellent functional and aesthetic outcomes when applied judiciously by experienced multidisciplinary teams [1,2,4,9,10]. The prevailing consensus favors individualized decision-making that accounts for patient age, anatomical characteristics, disease severity, reconstructive complexity, surgeon and institutional experience, and patient or family preferences, rather than adherence to a uniform timing algorithm [11,69].

In pediatric microtia patients, the timing of auricular reconstruction and hearing rehabilitation remains particularly controversial [11,69]. Traditional approaches defer auricular reconstruction until sufficient rib cartilage is available, typically between 8 and 10 years of age, potentially delaying aesthetic and functional rehabilitation during critical developmental periods [11]. Emerging strategies involving early hearing device implantation prior to auricular reconstruction address functional needs earlier but raise concerns regarding device longevity, the need for future upgrades and possible interference with later reconstructive procedures [9,10]. Management of unilateral microtia with contralateral normal hearing remains especially debated, as bilateral hearing confers advantages in sound localization and speech perception in noisy environments, yet the benefit–risk ratio of intervention in unilateral cases continues to be questioned [10,11,69]. Anesthetic considerations further influence decision-making, particularly in younger children. Leone et al. demonstrated the feasibility of minimally invasive Ponto surgery under local anesthesia in pediatric patients, potentially reducing anesthesia-related risks, although this approach requires patient cooperation and may not be suitable for all age groups [123].

**Figure 1:**
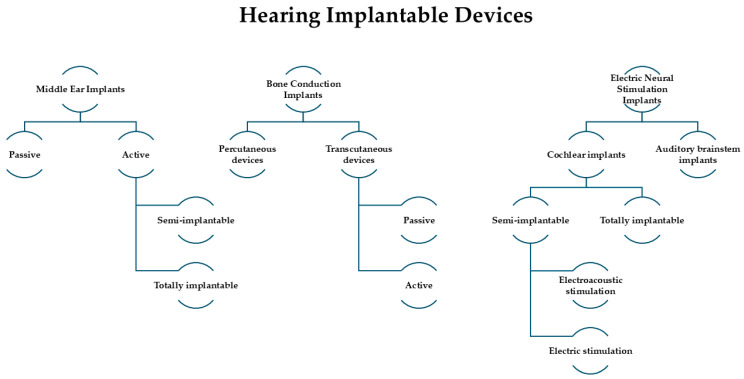
Classification of hearing implantable devices, adapted from Bruschini et al. [149] in accordance with the CC-BY-NC-ND (Creative Commons Attribution-NonCommercial-NoDerivatives 4.0 International) license.

Over the past several decades, substantial advances have led to the development of a broad spectrum of implantable hearing devices. Figure 1 offers an overview of these devices [149].

Comparative evaluation of bone conduction hearing devices has not demonstrated significant differences in hearing gain among Bonebridge, Sophono, and BAHA Attract systems in pediatric microtia patients. This finding suggests that device selection should incorporate considerations beyond audiological performance alone, including surgical approach, anatomical constraints, transcutaneous versus percutaneous design, MRI compatibility, economic factors, and patient or family preferences [3].

Kurihara et al. further examined the advantages and limitations of implanting bone conduction devices in the poorer-hearing ear of patients with asymmetric hearing loss, indicating that the implantation strategy itself may influence outcomes and warrants further investigation [150].

The choice between middle ear implants, such as the Vibrant Soundbridge, and bone conduction devices for conductive or mixed hearing loss remains debated. While middle ear implants may offer advantages in sound quality and avoidance of transcutaneous attenuation, they require suitable middle ear anatomy or access to the round window, whereas bone conduction devices offer broader applicability with simpler surgical techniques but may be limited in maximum output. Comparative effectiveness data remains limited, underscoring the need for further research in this area [9,10,109,149,150,151,152].

The Osia^®^ system is a newer-generation active transcutaneous bone conduction hearing implant that utilizes piezoelectric transduction to deliver mechanical vibrations directly to the cranial bone. By avoiding a percutaneous abutment, Osia aims to reduce soft tissue complications while maintaining effective sound transmission for patients with conductive or mixed hearing loss and single-sided deafness. Early clinical studies have reported favorable audiological outcomes and patient satisfaction, with particular strengths in mid-to-high frequency transmission; however, long-term data and direct comparative studies with established systems such as Bonebridge and BAHA Attract remain limited, precluding definitive conclusions regarding relative superiority [153,154,155,156].

### 8.2. Recent Innovations and Technological Advances

Technological advances continue to shape the field of auricular reconstruction and hearing rehabilitation. Bioprinting and tissue engineering hold promise for future auricular reconstruction by potentially eliminating donor-site morbidity associated with rib cartilage harvest, although clinical translation awaits further validation [11]. Ongoing refinement of imaging modalities, virtual surgical planning and intraoperative navigation is expected to improve precision and outcomes in complex reconstructions [11,26,133].

Concurrently, next-generation hearing devices aim to enhance auditory performance, reduce complication rates, improve MRI compatibility and further minimize surgical invasiveness, with fully implantable cochlear implants and advanced transcutaneous bone-conduction systems representing areas of active development [8,105].

Three-dimensional imaging and virtual surgical planning have become increasingly valuable tools in complex temporal reconstruction and hearing device implantation [11]. Della Volpe et al. described the use of three-dimensional reconstruction models to optimize Bonebridge placement in patients with severe aural atresia, allowing precise preoperative assessment of implant position relative to critical anatomical structures [133]. Similarly, Frisina et al. employed OTOPLAN software for preoperative electrode selection in a case of cochlear implantation complicated by temporal bone fracture and cochlear ossification, with virtual planning contributing to the selection of an appropriate surgical approach and successful clinical outcome [26]. The expanding availability of three-dimensional printed models for surgical training and operative planning represents a further advance, with emerging bioprinting technologies under investigation pending validation of safety and clinical effectiveness [11].

Minimally invasive surgical approaches have been developed with the aim of reducing soft tissue trauma, improving cosmetic outcomes and facilitating recovery while preserving functional results [122,157].

Minimally invasive Ponto surgery employs a punch incision technique with a dedicated drill guide, thereby limiting soft tissue dissection compared with traditional flap-based approaches. Leone et al. demonstrated the feasibility of this technique under local anesthesia in pediatric patients, achieving favorable hearing outcomes with minimal soft tissue complications, as well as potential advantages in operative time reduction and aesthetic results [123]. Additional efforts to reduce surgical invasiveness include minimal invasive pocket techniques for magnet-based bone-anchored hearing devices without fixation, reflecting ongoing refinement of implantation strategies despite the limited outcome data currently available.

Several novel surgical approaches have been introduced to address specific challenges associated with combined reconstruction and hearing rehabilitation. The retrosigmoid sinus approach for Bonebridge implantation represents a significant innovation for patients undergoing or having completed auricular reconstruction, as it positions the bone conduction floating mass transducer posterior to the sigmoid sinus and avoids interference with reconstructed auricular tissue or expanded postauricular flaps [4].

Wang et al. demonstrated that this approach does not increase surgical risk, compromise audiological efficacy, or significantly prolong anesthesia time when compared with conventional techniques [4].

Ueda et al. proposed a novel incision design for Vibrant Soundbridge implantation prior to auricular reconstruction, positioned approximately 5 cm posterior to the temporomandibular joint and at least 2 cm from the planned auricular reconstruction site, thereby allowing early hearing rehabilitation without compromising subsequent two-stage auriculoplasty [9]. Periosteal flap techniques have also been explored in cochlear implantation, with Kothandaraman et al. reporting preliminary experience, suggesting potential benefits for wound healing, although detailed outcome data remain limited [157].

Complex surgical prelamination techniques can be employed using cartilaginous frameworks, which may consist either of auricular fragments resulting from post-traumatic auricular amputations or autologous cartilaginous frameworks fashioned from costal cartilage. An alternative option is represented using a framework represented by porous polyethylene (Medpor^®^, Stryker; Kalamazoo, MI, USA) implants. These structures can be used for the prelamination of flaps that are subsequently transferred to the auricular region. Such an approach is particularly well-suited to radial forearm flaps, which are well known for their versatility and the advantages they offer in tissue prefabrication. These indications are especially relevant when local tissue resources do not allow for the creation of an adequate cutaneous and soft tissue envelope in the temporal region to accommodate a cartilaginous framework, in cases of extensive tissue destruction, or when local reconstructive options at this level have been exhausted [44,91,158,159,160].

Future directions should prioritize comparative effectiveness research, long-term outcome studies, pediatric-specific investigations and cost-effectiveness analyses to further refine clinical practice.

## 9. Conclusions

Microsurgical reconstruction of the ear and temporal region with integrated hearing rehabilitation represents a rapidly evolving field characterized by technological innovation, refined surgical techniques, and increasingly patient-centered approaches. Recent literature evidence demonstrates that both simultaneous and staged approaches can achieve excellent aesthetic and functional outcomes when performed by experienced multidisciplinary teams with appropriate patient selection. Further research is required in this field to optimize structural and functional outcomes, ensure long-term stability of results and achieve optimal quality of life.

The future direction of the field increasingly favors personalized treatment strategies that incorporate advanced imaging modalities, virtual surgical planning and patient-centered outcome measures to optimize both structural and functional results. As technological capabilities expand and the body of evidence grows, the integration of microsurgical reconstruction with auditory rehabilitation is expected to become more refined and better coordinated, enabling comprehensive restoration of both aesthetic integrity and hearing function in patients with auricular and temporal bone defects.

## Figures and Tables

**Table 1:** Congenital and Acquired Conditions Requiring Ear and Temporal Bone Reconstruction [1,2,3,6,13,14,15,16,17,18,19,20,21,22].

Category	Condition	Descriptive Features	Typical Hearing Status	Reconstructive Challenges	Hearing Rehabilitation Considerations
Congenital	Microtia/Aural atresia–Grade I (Weerda)	Recognizable auricle with preserved landmarks; EAC present but may be stenotic	Moderate conductive hearing loss	Relatively mild deformity; canal may be usable or require minor correction	Conventional or minimally invasive options may be possible depending on canal patency
	Microtia/Aural atresia–Grade II (Weerda)	Partially formed auricle; disorganized cartilage; rudimentary or absent EAC	Moderate conductive hearing loss	Increased complexity due to cartilage deficiency and absent canal	Bone conduction or implantable hearing solutions frequently required
	Microtia/Aural atresia–Grade III (Weerda)	Severely hypoplastic “peanut-shaped” remnant or anotia; complete aural atresia; middle ear malformations	Significant conductive hearing loss (often 50–60 dB)	Canaloplasty rarely feasible; total auricular reconstruction needed	Implantable hearing devices are typically the preferred strategy
Acquired	Temporal bone trauma	Composite defects of skin, soft tissue, and bone; possible EAC/middle or inner ear injury	Variable; may include conductive, sensorineural, or mixed loss	May involve CSF leak, facial nerve injury, cochlear damage; highly individualized reconstruction	Rehab depends on cochlear and nerve integrity; implantable devices often considered
	Chronic suppurative otitis media	Chronically inflamed middle ear and mastoid; poor tissue quality	Conductive or mixed hearing loss	Scarred, infected field increases complication risk	The middle ear often unsuitable for reconstruction; implantable options favored
	Cholesteatoma	Bone erosion; distorted anatomy from inflammation and prior surgery	Conductive or mixed hearing loss	Limited healthy tissue; recurrence risk	Non-ear-canal-dependent hearing solutions commonly required
	Malignancy	Composite loss of skin, bone, EAC, and middle ear after oncologic resection; various tumors starting from the skin, external auditory canal, middle ear, temporal bone, parotid gland, cranial nerves, or intracranial structures	Usually severe conductive or mixed loss	Requires lateral/subtotal temporal bone resection; need for cranial coverage and protection of vital structures	Hearing rehabilitation secondary to oncologic safety; implantable devices are considered when feasible
	Refractory chronic otitis media/advanced cholesteatoma requiring subtotal petrosectomy	Surgical closure of EAC, Eustachian tube obliteration, cavity obliteration	Native conductive pathway eliminated	Major ablative procedure but creates a stable, infection-free cavity	Provides an optimal environment for simultaneous or staged implantable hearing rehabilitation

**Table 2:** Complications associated with microsurgical ear and temporal region reconstruction with hearing rehabilitation [1,3,4,8,16,105,114,115,116,117,118,119,120,121,122,123].

Category	Complication	Description	Management
Surgical at recipient site	Infection	More frequent in combined or single-stage procedures, especially when cavity obliteration and implantation are performed simultaneously	Antibiotic therapy, surgical debridement, device removal if necessary, staged reimplantation
	Wound dehiscence/delayed healing	Increased risk in previously operated, irradiated, or infected fields	Local wound care, secondary closure, revision surgery in selected cases
	Hematoma/seroma	Common minor complication, especially after combined auricular reconstruction and implantation	Conservative treatment, aspiration if required
	Cerebrospinal fluid (CSF) leak	Dural violation or inadequate sealing of the otic capsule, particularly in subtotal petrosectomy	Multilayer closure, fibrin sealants, lumbar drainage, revision surgery in persistent cases
	Vascular injury (sigmoid sinus, jugular bulb)	Extensive temporal bone drilling, altered anatomy	Immediate intraoperative control, packing, repair
Neurological	Facial nerve injury	Direct trauma, thermal injury, traction, ischemia during tumor surgery or implantation in malformed temporal bones	Immediate repair (end-to-end anastomosis or nerve grafting), corticosteroids for delayed palsy, secondary facial reanimation if needed
	Delayed facial palsy	Inflammatory or viral mechanisms	Conservative treatment, corticosteroids, observation
Device-related	Skin reactions (percutaneous systems)	Soft tissue complications around abutment site	Local care, antibiotics, revision if persistent
	Pressure-related erythema (transcutaneous systems)	Excessive magnetic coupling	Temporary device discontinuation, magnet strength adjustment
	Implant extrusion (theoretical)	Insufficient soft-tissue coverage, infection	Surgical revision, implant removal if required
	Incomplete electrode insertion (cochlear implant)	Cochlear ossification, anatomical distortion, post-tumor surgery	Acceptance of partial insertion, revision in selected cases
	MRI interference (cochlear implant)	Tumor-related follow-up imaging limitations	Careful preoperative selection, alternative surveillance strategies
Flap-related	Venous congestion/arterial thrombosis	Microsurgical failure, early postoperative period	Urgent surgical re-exploration
	Partial flap necrosis	Marginal perfusion compromise	Local wound care, minor revision
	Total flap loss	Rare, more frequent in high-risk or revision cases	Secondary reconstruction
	Contour irregularities/fat necrosis	Flap volume mismatch or ischemia	Secondary revision procedures
Donor site	Hematoma/seroma	Flap harvest-related	Conservative management, drainage
	Infection/delayed healing	Patient factors, flap type	Local wound care, antibiotics
	Sensory disturbance	Nerve sacrifice or traction	Observation, usually self-limited
	Aesthetic dissatisfaction	Visible scars or contour changes	Patient counseling, secondary revision if needed
Revision surgery	Device failure or osseointegration failure	Implant-specific or biological factors	Revision fixation or device upgrade
	Infection-related revision	More frequent in single-stage complex procedures	Staged revision, delayed reimplantation

**Table 3:** Consensus and controversies regarding reconstructive strategies of the auricular region and auditory rehabilitation [1,3,4,9,10,69,101,140,141,142,143,144,145,146,147,148].

Overview of Existing Knowledge	Aspect	Summary	Available Evidence	Representative Studies Exemplified
Consensus	Safety and efficacy of implantable hearing systems	Bone conduction devices and other implantable hearing devices provide meaningful hearing benefit in appropriately selected patients with conductive/mixed loss or auricular malformations.	Systematic review/meta-analysis; retrospective cohorts	Magele et al., 2019: Systematic review of 39 studies (487 patients) evaluating active transcutaneous bone conduction implants. Meta-analysis showed a mean functional gain of 30.89 dB (95% CI 27.53–34.24). Mean weighted improvement in word recognition score at 65 dB was 52.1%. Among 286 implanted ears, 90.6% had no complications; minor adverse events occurred at a rate of 1 per 9.9 person-years and major adverse events at 1 per 148.9 person-years [143]. Caversaccio et al., 2025: Systematic review/meta-analysis of 170 articles on 6451 implanted ears found that after adjustment to 12-month follow-up, pooled minor event rates per 100 patients/year were 9.1 for aBCIem, 25.8 for aBCIpz, 33.8 for tBAHA, and 34.7 for pBAHA; major event rates were 3.3, 5.6, 3.0, and 13.2, respectively. Revision surgery rates were 1.7, 3.0, 1.6, and 9.4 per 100 patients/year, and non-user rates were 1.3, 0.7, 6.5, and 1.1, respectively. Follow-up time significantly affected incidence rates (*p* < 0.001); aBCIem had the lowest minor complication rate, pBAHA had the highest major event and revision surgery rates, and tBAHA had the highest non-user rate [144].
	Multidisciplinary/integrated planning	Reconstruction and hearing rehabilitation should be coordinated rather than planned independently.	Expert consensus/integrated management recommendations	Truong et al., 2022: national expert working group of 9 experts on microtia and atresia. This guideline emphasizes coordinated management of hearing rehabilitation, hearing reconstruction, and auricular reconstruction in patients with microtia and aural atresia. Close communication between the atresia/hearing surgeon and the microtia reconstruction surgeon is recommended to develop a cohesive treatment timeline and optimize outcomes, recognizing that each patient requires an individualized management plan [68].
	Feasibility of simultaneous reconstruction + bone-conduction implantation	Simultaneous reconstruction and bone conduction implantation is feasible in selected patients in experienced centers.	Retrospective single-center case series and studies	Wang et al., 2018: 7 bilateral microtia patients (7–11 years) underwent two-stage auricular reconstruction with simultaneous BoneBridge implantation during stage II; mean auditory threshold improvement 34.8 dB HL, with no complications reported [145]. Chen et al., 2025: 41 bilateral microtia patients underwent combined auricular reconstruction and bone conduction device implantation (Baha Attract *n* = 13, Sophono *n* = 18, Bonebridge *n* = 10), demonstrating significant improvement in aided hearing thresholds and speech recognition without major adverse events [3]. Lv et al., 2025: Retrospective study of 44 patients with bilateral microtia undergoing simultaneous auricle reconstruction and BONEBRIDGE implantation via the retrosigmoid sinus approach showed stable bone conduction thresholds postoperatively and a 27.2 dB HL improvement in aided sound-field thresholds. Speech recognition in quiet environment improved to 72% for monosyllables, 84% for disyllables, and 98% for short sentences. During 8–51 months of follow-up, audiological outcomes remained stable, with no significant complications reported, and all patients were satisfied with the aesthetic results of auricular reconstruction, indicating that the retrosigmoid approach is a safe and effective option when combined with auricular reconstruction [146].
Controversies	Timing of procedures (simultaneous vs. staged)	No high-level comparative evidence definitively establishes superiority of simultaneous or staged strategies for auricular reconstruction plus implantable hearing rehabilitation.	Mainly retrospective series/case reports	Evidence regarding the optimal timing of reconstruction and hearing implantation is primarily derived from small case series and case reports, including Wang et al., 2018 (*n* = 7); Ueda et al., 2025 (*n* = 4); and Yoshimura et al., 2021 (single case). These studies demonstrate the feasibility of both simultaneous and staged approaches; however, the overall evidence base remains limited and heterogeneous, supporting an individualized, patient-specific approach to procedural timing [9,10,145].
	Implantation before later auricular reconstruction	Early hearing rehabilitation before later auricular reconstruction may be feasible, but evidence is limited.	Small retrospective series and case report	Ueda et al., 2025: four patients with unilateral microtia underwent Vibrant Soundbridge (VSB) implantation prior to subsequent auricular reconstruction; all later underwent successful auricular reconstruction with preservation of auricular contour and no deterioration in hearing outcomes. Yoshimura et al., 2021: single case report describing VSB implantation before later auricular reconstruction in a child with unilateral microtia-atresia, demonstrating the feasibility of this staged approach [9,10].
	Device selection	Audiological outcomes across bone conduction systems appear broadly comparable, shifting device selection toward anatomical factors, surgical considerations and patient preference.	Comparative cohort studies and systematic reviews	Chen et al., 2025: retrospective cohort of 41 bilateral microtia patients comparing Baha Attract (*n* = 13), Sophono (*n* = 18), and Bonebridge (*n* = 10) in the context of combined auricular reconstruction and hearing implantation, demonstrating significant aided hearing improvement with no significant inter-group differences in hearing gain and only minor device-related complications managed conservatively [3]. Shoman et al., 2022: pediatric multicenter comparative cohort (18 Bonebridge vs. 8 Baha Attract) showing effective hearing improvement with both active and passive transcutaneous systems, although active devices demonstrated superior performance at higher frequencies; the cohort included mixed etiologies and was not limited to microtia [147]. Brunner et al., 2024: systematic review with indirect treatment comparison based on three studies (80 Osia vs. 54 Baha Attract patients) reporting superior outcomes for Osia in PTA4, speech perception in quiet and noise, and a clinically meaningful HUI3 (Health Utilities Index Mark 3) utility benefit (+0.03) with Osia, with at least equivalent safety; however, the evidence is indirect, not microtia-specific, and should be interpreted cautiously [148].
	Complication profiles	In complex infected or anatomically unfavorable ears requiring subtotal petrosectomy, complication and revision burdens remain clinically relevant, particularly for cochlear implantation in selected high-risk settings. By contrast, simultaneous auricular reconstruction with bone conduction devices in microtia series has generally shown lower rates of major complications, although these comparisons are indirect and based on different patient populations	Systematic review/meta-analysis and retrospective institutional cohort	Yan et al., 2020: systematic review and meta-analysis including 27 studies (377 patients, 397 subtotal petrosectomy procedures); reported an overall complication rate of 12.4% and cholesteatoma recidivism of 9.3%, with no significant difference in complication rates between single-stage and multistage procedures, although multistage surgery was more frequently employed in cholesteatoma cases (42.6% vs. 20.1%) [101]. Zhang et al., 2021: retrospective single-institution cohort of 32 patients undergoing 37 subtotal petrosectomy procedures (33 cochlear implants, 4 osseointegrated hearing implants); 6 procedures (16%) required revision surgery due to refractory postauricular infection or wound breakdown, whereas no revision procedures occurred in patients receiving osseointegrated hearing implants [1].
	Economic evidence	Cost-effectiveness evidence remains limited and device-specific rather than reconstruction-strategy-specific.	Indirect treatment comparison and model-based economic evaluation	Brunner et al., 2024: systematic literature review and indirect treatment comparison based on three prospective studies (80 Osia vs. 54 Baha Attract patients) followed by a Markov cost-utility model. The analysis reported superior audiological outcomes and a clinically meaningful HUI3 utility gain (~0.03) with the Osia system and estimated an ICER of ~29,301 Australian dollars per QALY gained, indicating cost-effectiveness versus Baha Attract in the Australian healthcare setting. However, the evidence is indirect, model-based, and not specific to microtia or reconstructive timing strategies [148].

## Data Availability

No new data were created.
